# Towards Personalized Intervention for Alzheimer’s Disease

**DOI:** 10.1016/j.gpb.2016.01.006

**Published:** 2016-09-28

**Authors:** Xing Peng, Peiqi Xing, Xiuhui Li, Ying Qian, Fuhai Song, Zhouxian Bai, Guangchun Han, Hongxing Lei

**Affiliations:** 1 CAS Key Laboratory of Genome Sciences and Information, Beijing Institute of Genomics, Chinese Academy of Sciences, Beijing 100101, China; 2 Cunji Medical School, University of Chinese Academy of Sciences, Beijing 100049, China; 3 Center of Alzheimer’s Disease, Beijing Institute for Brain Disorders, Beijing 100053, China

**Keywords:** Alzheimer’s disease, Demographic information, Genome, Peripheral biomarkers, iPSC technology

## Abstract

**Alzheimer’s disease** (AD) remains to be a grand challenge for the international community despite over a century of exploration. A key factor likely accounting for such a situation is the vast heterogeneity in the disease etiology, which involves very complex and divergent pathways. Therefore, intervention strategies shall be tailored for subgroups of AD patients. Both demographic and in-depth information is needed for patient stratification. The **demographic information** includes primarily *APOE* genotype, age, gender, education, environmental exposure, life style, and medical history, whereas in-depth information stems from **genome** sequencing, brain imaging, **peripheral biomarkers**, and even functional assays on neurons derived from patient-specific induced pluripotent cells (iPSCs). Comprehensive information collection, better understanding of the disease mechanisms, and diversified strategies of drug development would help with more effective intervention in the foreseeable future.

## Introduction

Alzheimer’s disease (AD) is one of the leading causes of death in senior people. Caring for AD patients with deteriorating cognitive and daily functions poses a great economic and psychological burden for the families as well as society. Initially discovered in 1906, the pathological hallmarks of AD, namely amyloid plaques and neurofibrillary tangles, have been well documented over a century. However, little had been known about the disease mechanisms at molecular level until the identification of the gene encoding amyloid precursor protein (*APP*) and the genetic mutations causing familial AD [1], [2]. Nonetheless, familial AD only constitutes ∼2% of AD patients [3], while the vast majority of AD cases are not caused by the genetic mutations affecting the coding or processing of *APP*. For sporadic AD, the molecular pathogenesis seems to be far from understood as yet.

Up till now, only four drugs have been approved for AD treatment by the Food and Drug Administration (FDA) in USA and its counterparts in Europe [4]. These drugs target neural transmission and are all used for symptom relief, sometimes even with unbearable side effects. They are unable to modify the disease trajectory, not even slowing down the disease progression. Therefore, the “neural transmission” hypothesis for AD has not been well supported by the human trials, and deficiency in neural transmission may merely be a downstream and symptomatic problem in AD. In the past couple of decades, most of the efforts on drug development have been devoted to the clearance of amyloid or the aggregating oligomers, which is believed to be a major causal factor according to the “amyloid cascade hypothesis” [5]. Although recent studies targeting amyloid showed marginal progress in the early stage AD [6], [7], most of the clinical trials along this line have been very disappointing. Beyond amyloid clearance, other prevention or treatment strategies have also been initiated with no conclusive evidence of success so far [8].

**Figure 1:**
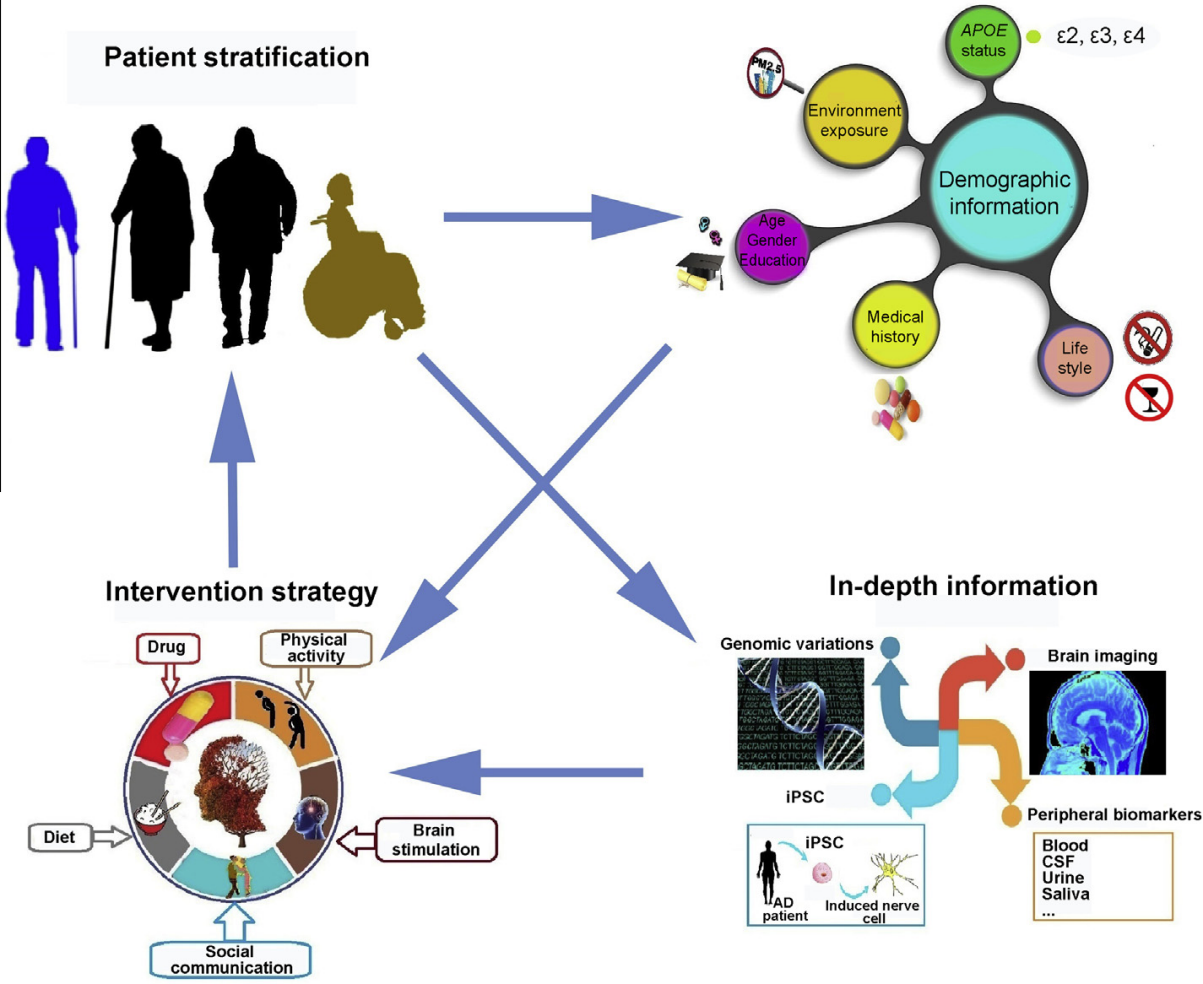
**Path from patient stratification to personalized intervention for AD** Collection of demographic information is the basis for patient stratification. Incorporation of in-depth information will greatly facilitate the design of personalized intervention. AD, Alzheimer’s disease; iPSC, induced pluripotent stem cell; CSF, cerebrospinal fluid; PM 2.5, particulate matter (⩽2.5 μm).

To achieve more positive outcome, better patient stratification shall be adopted in the future based on comprehensive collection of patient information. The details will be discussed in the following sections (Figure 1).

## Demographic information for AD

Currently, the well-recognized demographic information about AD includes *APOE* genotype, age, gender, education, environmental exposure, life style, and medical history.

### *APOE* genotype

*APOE* ɛ4 allele is the major genetic risk of sporadic AD, which confers risks 3–4 folds higher for people carrying one ɛ4 allele and ∼10 folds higher for peoples carrying two ɛ4 alleles compared to non-carriers [2]. Although mainly viewed as a gene involved in the lipid metabolism pathway, *APOE* seems to be associated with many AD-related processes. Most notably, *APOE* is involved in amyloid β (Aβ) metabolism and amyloid clearance [9]. *APOE* may also be involved in brain development. For example, infants carrying *APOE* ɛ4 allele have different brain structure compared to non-carriers [10]. The altered brain structures at early developmental stage may confer susceptibility for AD at the old age. *APOE* ɛ4 genotype is also associated with reduced glucose metabolism in the brain independent of amyloid aggregation [11]. Since deficiency in energy metabolism is considered as one of the major upstream factors in AD pathogenesis, this study suggests that *APOE* genotype itself can be a causal factor for AD. Thus, AD patients with 0, 1, or 2 ɛ4 alleles may follow different paths to the disease stage and therefore shall be treated differently. Due to the critical roles of *APOE* in the development of sporadic AD, *APOE*-centered drug development has also been attempted, and notably drugs targeting *APOE* have demonstrated efficiency in mouse model of AD [12].

### Age

Apart from *APOE*, age is the most recognized risk factor for sporadic AD. Sporadic AD mostly starts after the age of 65, while the chance of developing AD climbs to ∼40% for people aged over 85 [13]. Aging is accompanied by the systematic deterioration of the condition over the whole body. Most notably, brain perfusion (blood supply to the whole brain) continues to decline starting at the age of 22, which is likely the root cause of many problems due to the critical role of energy supply in brain functions. Studies have shown that brain atrophy and amyloid deposition are part of the normal aging process. Even in normal elderly people without dementia, over 1/3 has amyloid plaque in the brain [14]. In addition, brain transcriptome studies showed that the aging brain is characterized by up-regulation of immune response and down-regulation of synaptic transmission, which is similar to the gene dysregulation pattern in AD [15]. To some extent, AD can be viewed as accelerated aging. Interestingly, aging is lately considered by some people as a treatable disease and anti-aging clinical trials have been proposed [16].

### Gender

Epidemiological studies suggest that females have a higher risk of developing AD than males do [17], although the exact reason remains unclear at this moment. It has been postulated that decreased estrogen levels at old age may be partially responsible for higher incidence of AD in females [18]. Estrogen is neuroprotective and estrogen receptor β plays a critical role in brain function [19]. Studies on the human brain transcriptome have also shown that immune response is more widely spread in aged female brains than aged male brains, consistent with the accelerated aging scenario in female brains [20]. There is interaction between gender and *APOE* in both brain connection and cerebrospinal fluid (CSF) markers [21], which may be partially attributed to the involvement of both estrogen receptor β and *APOE* in certain brain functions. Further elucidation of the gender risk of AD may require better understanding of the brain network through ongoing big brain projects worldwide.

### Education

Lower education level is associated with higher risk of developing AD [22]. Education process is accompanied by sophisticated brain wiring. Lower education likely leads to less complex brain network which is more susceptible for breakdown, which is the so-called “brain reserve” hypothesis [23]. However, it has also been reported that the rate of cognitive decline is higher for people with higher education once AD is diagnosed [24]. This is likely due to the depletion of “brain reserve” after the onset of AD symptoms even for people with higher education.

### Environmental exposure

Exposure to non-healthy environment is also associated with higher risk of developing AD. For example, long-term exposure to high concentration of fine particulate matter (⩽2.5 μm) environment can lead to peripheral biomarker change linked to AD [25]. Mechanistic studies have shown that similar environment can lead to elevated inflammation and AD pathologies in mice [26]. Additionally, high concentration of pesticide, such as dichlorodiphenyldichloroethylene (DDE), in the blood is also associated with higher AD risk [27]. Overall, environmental exposure may affect the brain either directly through the inhalation process or indirectly through the blood–brain barrier [25].

### Life style

Life style can have major effects on the risk of developing AD. Cigarette smoking and alcohol drinking are associated with higher risk, both of which may disrupt the global metabolism and in the meantime exert epigenetic modification in the brain [28]. On the other hand, more active lifestyle, such as physical exercise [29], social communication, and brain stimulating activities like music and arts [30], has a protective effect. The beneficial effect may arise from better energy supply and more synaptic activities.

### Medical history

Certain medical histories are associated with elevated AD risk. For instance, hypertension is associated with both vascular dementia and AD [31], which is consistent with the critical role of the cardiovascular system and energy supply in AD development. In a population-based study over 25 years, late-life atrial fibrillation was found to be the highest risk of AD among several heart diseases examined [32]. In another longitudinal study, the duration of type 2 diabetes was found to be a risk factor for AD [33]. In addition, periodontal disease is also associated with higher risk of AD through increased brain amyloid load [34]. In a meta-analysis over studies on the relationship between bacterial infection and AD, certain bacterial infection such as by *Chlamydophila pneumoniae* was found to increase the risk of 4–10 fold, likely through sustained high level of immune response [35]. Notably sleep has been recently linked to AD because of its role in amyloid clearance [36], [37], and sleep postures may affect efficiency in amyloid removal [38].

## In-depth information for AD

Demographic information alone is insufficient to achieve precise assessment of AD patients, which would be greatly facilitated by collecting in-depth information including genome, brain imaging, and peripheral biomarkers. In addition, recent development in induced pluripotent stem cell (iPSC) technology makes it possible to conduct functional assays on patient-derived neurons.

### Genomic variations

Although *APOE* ɛ4 allele confers high risk of developing AD, it is neither necessary nor sufficient for AD development [39]. For instance, some people without *APOE* ɛ4 alleles can develop AD, while others carrying one or two *APOE* ɛ4 alleles may never develop AD throughout their lives. Genome-wide association studies (GWAS) have revealed 20 additional risk loci such as those encoding clusterin (*CLU*) and bridging integrator 1 (*BIN1*), all with moderate risk [40]. A recent work showed that marginally-significant SNPs (*P* < 10^−3^) from GWAS studies may contribute significantly to the prediction of genetic risk of AD [41]. Other than the genetic risk from common variations examined in GWAS studies, rare variations detected by sequencing technologies may also confer risk of AD. For instance, rare variations in genes encoding triggering receptor expressed on myeloid cells 2 (*TREM2*) [42] and phospholipase D family, member 3 (*PLD3*) [43] from exome sequencing studies could significantly increase the risk of developing AD, albeit limited to a small population of the rare variant carriers. The relative contribution from common and rare variations is still under debate, which could be tackled using whole-genome sequencing (WGS) since compared to GWAS, WGS can detect both common and rare variations once sufficient sequencing depth is reached.

### Brain imaging

Brain imaging offers the direct measurement of the patients’ brain structure or function. The degree of brain atrophy can be evaluated by magnetic resonance imaging (MRI). Glucose uptake as an indicator of energy metabolism level can be measured by 18F-fluorodeoxiglucouse positron emission tomography (PET). For more specific diagnosis of AD, various PET technologies such as Pittsburgh compound B (PIB) have been developed for the measurement of Aβ amyloid [44] and tangle [45], [46]. PET and MRI have also been combined to elucidate the causal relationship between amyloid deposition and regional cerebral blood flow [47].

### Peripheral biomarkers

Brain imaging is currently not suitable for early diagnosis due to its poor accessibility especially in China. Therefore, peripheral biomarkers from CSF, blood, urine [48], and saliva [49] are highly desirable. Most notably, low level of Aβ42 and high level of p-tau in CSF have been demonstrated to be sensitive biomarkers for AD diagnosis [50]. Aside from these well-recognized biomarkers, other biomarkers, mostly in the peripheral blood, have also been investigated. For example, the expression levels of several microRNAs including miR-15a in the blood are associated with AD [51]. Lower expression level of ribosome genes has also been reported in the blood of AD patients [52]. Apart from gene expression, the level of immune-related proteins or analytes in the plasma or serum has also been associated with AD. For example, complement C3 has been replicated in independent studies [53]. A panel of 21 serum proteins has also been replicated in independent sample sets [54]. A panel of blood analytes including pancreatic polypeptide (PPY) shows high correlation with brain amyloid load [55]. In addition, a protein microarray study identified a panel of 20 autoantibodies with high accuracy in discriminating AD from control [56]. A multi-tissue study showed that calpain activity is increased in CSF and decreased in blood of AD patients [57]. Interestingly, a rare study of telomere in buccal cells showed shorter telomeres and increased number of telomeres in AD patients compared to controls [58].

### Functional assays on patient-derived neurons

The most relevant cell type for AD studies is neuron, which is not accessible in patients. Notably, the development of iPSC technology opens the door to examine patient-specific neurons derived from the skin fibroblast. Various assays can be conducted on these neurons. For instance, Aβ42/Aβ40 ratio and gene expression profiles were compared on iPSC-derived neural progenitor cells from *PSEN1* mutation carriers and controls [59]. A *PSEN1* ΔE9 mutation was shown to specifically affect the γ-secretase activity of *PSEN1* [60]. Neurotoxicity caused by various forms of Aβ can be examined too [61]. Moreover, these neurons can also be used to test whether drugs like γ-secretase modulators are effective in reversing certain cellular phenotypes [62]. In the past few years, 3D culture models have been under development, which may better model the 3D neuronal network *in vitro* [63].

## Intervention strategies for AD

Although there are limited options available for treating AD, proper intervention as discussed in this section is highly recommended. Prevention is the best medicine [64]. Therefore, it is a common view to treat the disease at its early stage, with or without medication.

### Physical activity

The effect of physical exercises on AD has been extensively studied in both human and mouse models, with the beneficial effect reported at different stages of AD. For example, regular physical exercises can lower the rate of conversion from mild cognitive impairment (MCI) to dementia [65]. The exact mechanism underlying such beneficial effect is likely very complex. Obviously, exercise can improve the cardiovascular functions and provide better blood supply to the brain, whereas at the molecular level, it may involve elevated level of *BDNF* in the serum [66]. Interestingly, conventional exercises such as Tai-Chi have also been demonstrated to confer a wide spectrum of beneficial effects [67].

### Brain stimulation

Proper brain stimulation such as music is protective for AD. It may help maintain existing neural networks or develop new ones [68]. Music and arts are the common approaches for brain stimulation. Studies showed that certain sound waves may help clear amyloid from the blood stream [69]. In addition, non-invasive stimulations such as transcranial magnetic stimulation can improve cognition [70]. To restore certain neuronal circuits in patients with confirmed neurodegeneration, invasive deep brain stimulation has been tested in AD and Parkinson’s disease [71]. To reduce the side effects from invasive operation, some minimally invasive brain stimulation approaches are also currently under development [72].

### Social communication

Social communication can improve cognitive function for AD patients. Active cognitive life style such as social engagement has been shown to be associated with reduced risk of developing AD [73], whereas elderly people living a solitary life tend to have higher risk of developing depression [74], which is associated with higher risk of AD [75]. Thus, engaging elderly people in community activities is strongly recommended.

### Diet

Western diet is associated with higher risk of developing AD, while Mediterranean diet is protective [76], [77]. Moreover, Mediterranean diet and physical activity are independently protective for AD [29]. The Mediterranean diet likely favors a stronger cardiovascular system which can provide better energy and nutrient supply to the brain. In addition, dietary supplements have also been widely studied. For example, omega3 has been shown by many studies to slow down cognitive decline [78] and consumption of cocoa flavanol can improve cognitive function [79]. Other than these supplements, Selenium as an anti-aging nutrient is also widely studied in the field of aging and AD [80].

### Drugs

The four FDA-approved drugs for AD mainly target synaptic stimulation. In the past few years, the most actively pursued drug development for AD is Aβ immunotherapy that targets the clearance of amyloid plaques or Aβ oligomers [6]. Along the same line, targeting the pathological processing of Aβ has also been pursued, mainly focusing on γ-secretase [81]. Tau, another major hallmark of AD, is also an actively-pursued drug target [82]. Clinical trials for drugs targeting γ-secretase or tau have not been successful so far [83], [84]. While these drugs target specific molecules, Chinese traditional medicine likely has broader impact on the brain and the body as a whole. For example, some of the widely-used Chinese traditional medicine for AD such as ginseng can stimulate blood flow in the brain [85], while some traditional Chinese medicine may also enhance amyloid clearance [86].

## Stage-specific intervention strategy for AD

Based on current findings, we here summarize a stage-specific intervention strategy for AD [87], [88]. Please note that some of the intervention approaches may be applicable to other stages as well (Table 1).

### Preimplantation stage

Intervention at the preimplantation stage is highly controversial due to the ethical issues. Currently, preimplantation diagnosis is only available for families with inheritable diseases [89], including familiar AD. It is foreseeable that genome editing may be used to correct the causal mutations in familiar AD in the future. However, genome editing is not yet a mature technology for clinical practice at this moment due to the concerns including off-targeting. In addition, the ethical issue in human germline genome editing is extremely sensitive due to the risk of “playing God” by some people.

### Childhood, youth, and adulthood

Proactive prevention could be performed at stages of childhood, youth, and adulthood. This is particularly important for people who carry *APOE* ɛ4 allele or other high risk genetic variations. A good lifelong education can also help build a resilient brain network [24]. Furthermore, it is necessary to avoid non-healthy living and working environment. It is equally important to stick to a healthy diet and stay away from bad habits such as cigarette smoking and alcohol drinking.

### MCI and preclinical AD

With confirmed diagnosis from cognitive tests, peripheral biomarkers, or brain imaging, proper intervention shall be applied for MCI and preclinical AD. A good starting point is to have regular physical exercise. This can be supplemented with brain stimulating activities such as music and arts. Early stage patients should be actively involved in social communication with family, friends, or local community. Certain Chinese traditional medicine may also be helpful [86]. In addition, iPSC technology may be needed to properly design personalized treatment approaches.

### Mild AD

Once the patients progress to the clinical AD stage, medical intervention shall be added on top of the non-medical treatments described above. Several factors shall be taken into account for such intervention, including neural transmission, energy supply, and amyloid clearance. While prescribed drugs can stimulate neural transmission, nutritional supplements such as coenzyme Q10 may provide more energy supply to the brain [90]. Furthermore, the most heavily invested Aβ immunotherapy can remove the pathological amyloid buildup [6].

### Moderate AD

At the moderate AD stage, neural inflammation caused by prolonged stimulation by amyloid deposit may be a more pressing issue to deal with [91]. AD brain and blood transcriptome studies suggest that elevated level of the innate immune system may be a suitable target for further drug development [92]. For example, glucogon-like peptide 1 analogs, the widely-studied neuro-protective agent, are involved in reducing neural inflammation among many other protective functions [93]. More studies directly targeting neural inflammation including with non-steroidal anti-inflammatory drugs are underway [94].

### Severe AD

At the late stage of the disease, neuronal cell death becomes a major issue [95]. Neuronal cell death results in the permanent breakdown of the brain network, which is responsible for cognitive and daily functions. Thus, neural regeneration therapy could be more effective for patients at this stage. Interestingly, a recent study demonstrated enhanced learning and memory in AD mice after transplantation of inhibitory interneuron progenitors in the brain [96]. Stem cell technology will likely play an increasingly important role in neural regeneration. In addition to stem cell transplantation, activation of endogenous neural stem cells or converting the inflammatory astrocytes to neurons are other viable options [97]. However, these studies are still at the preclinical stages and the efficacy on human AD is thus unknown as yet.

## Concluding remarks

Although convergent AD symptoms have been observed, the underlying heterogeneous pathways for AD development require personalized intervention strategy. Adjustment of life styles may be a lifelong battle especially for those who carry causal mutations for familial AD or high risk genetic variations for sporadic AD. The combination of proper education, diet, life style, and environment seems to be the best prescription to prevent or delay the onset of AD. For patients at the early stage of the disease, physical and mental exercises, social communication, and traditional Chinese medicine could be effective to slow down the disease progression. For patients at the mild or moderate clinical AD stage, medical interventions, including those targeting neural transmission, energy supply, Aβ aggregation, and neural inflammation, should come into play. For patients at the severe stage with considerable loss of neurons, neural regeneration therapy could be critical to restore the normal function of the brain, although clinical trials have not been reported.

At each of the disease stages, intervention can be further tailored toward the patients according to the demographic and in-depth information described in this review. For example, drugs especially *APOE*-targeting drugs may act differently in patients with different *APOE* genotypes. As more drugs become available, it may be necessary to test the drug efficiency on patients with different genotypes. For such kind of test, patient-derived neurons can be used for preliminary tests. Other than the demographic and genetic factors, brain imaging and peripheral biomarkers may also be employed for patient stratification. In addition, age factor shall be considered when designing programs of physical activities because some activities may not be suitable for the highly-aged patients. For elderly females, hormone replacement therapy may be beneficial as well. Furthermore, for people with specific medical conditions such as hypertension or type 2 diabetes, management of these specific medical conditions is good for both AD prevention and treatment.

## Competing interest

The authors declare that they have no competing interests.

## Figures and Tables

**Table 1:** Stage-specific intervention strategies for AD

*Note:* AD, Alzheimer’s disease; Aβ, Amyloid beta; EOAD, early-onset Alzheimer’s disease; APOE, apolipoprotein E; iPSC, induced pluripotent stem cell.
